# Multiparametric MRI-based radiomics approach with deep transfer learning for preoperative prediction of Ki-67 status in sinonasal squamous cell carcinoma

**DOI:** 10.3389/fonc.2024.1305836

**Published:** 2024-06-13

**Authors:** Naier Lin, Yiqian Shi, Min Ye, Luxi Wang, Yan Sha

**Affiliations:** ^1^ Department of Radiology, Eye & ENT Hospital, Fudan University, Shanghai, China; ^2^ Department of Pathology, Eye & ENT Hospital, Fudan University, Shanghai, China

**Keywords:** sinonasal squamous cell carcinoma, Ki-67, deep transfer learning, radiomics, MRI

## Abstract

**Purpose:**

Based on comparison of different machine learning (ML) models, we developed the model that integrates traditional hand-crafted (HC) features and ResNet50 network-based deep transfer learning (DTL) features from multiparametric MRI to predict Ki-67 status in sinonasal squamous cell carcinoma (SNSCC).

**Methods:**

Two hundred thirty-one SNSCC patients were retrospectively reviewed [training cohort (n = 185), test cohort (n = 46)]. Pathological grade, clinical, and MRI characteristics were analyzed to choose the independent predictor. HC and DTL radiomics features were extracted from fat-saturated T2-weighted imaging, contrast-enhanced T1-weighted imaging, and apparent diffusion coefficient map. Then, HC and DTL features were fused to formulate the deep learning-based radiomics (DLR) features. After feature selection and radiomics signature (RS) building, we compared the predictive ability of RS-HC, RS-DTL, and RS-DLR.

**Results:**

No independent predictors were found based on pathological, clinical, and MRI characteristics. After feature selection, 42 HC and 10 DTL radiomics features were retained. The support vector machine (SVM), LightGBM, and ExtraTrees (ET) were the best classifier for RS-HC, RS-DTL, and RS-DLR. In the training cohort, the predictive ability of RS-DLR was significantly better than those of RS-DTL and RS-HC (p< 0.050); in the test set, the area under curve (AUC) of RS-DLR (AUC = 0.817) was also the highest, but there was no significant difference of the performance between DLR-RS and HC-RS.

**Conclusions:**

Both the HC and DLR model showed favorable predictive efficacy for Ki-67 expression in patients with SNSCC. Especially, the RS-DLR model represented an opportunity to advance the prediction ability.

## 1 Introduction

Sinonasal carcinomas are rare and aggressive neoplasms, accounting for approximately 3% of head and neck cancers (1), with sinonasal squamous cell carcinoma (SNSCC) representing the majority of cases (2, 3). As the clinical symptoms of SNSCC are often less marked and specific, many patients are diagnosed at advanced stages and associated with a poor prognosis (4).

The expression of Ki-67 protein has been widely used as an independent prognostic indicator in many malignant tumors. Numerous studies (5, 6) have proposed that a high level of Ki-67 status often indicates a more active cell proliferation, higher degree of aggressiveness (such as advanced tumor stage), and poorer prognosis. In sinonasal carcinomas, some literatures (7, 8) have demonstrated that patients with a high Ki-67 expression level (>50% positivity) tend to present a shorter 5-year disease-free survival, a higher possibility of local recurrence, and distant metastasis. According to these findings, the cutoff value of 50% for Ki-67 status was widely chosen as an optimal indicator for forecasting the outcome of patients with sinonasal neoplasms.

In clinical application, the Ki-67 status preoperatively is usually determined by immunohistochemistry methods from biopsy examination. However, as an invasive way, it is impossible to make accurate determination of the Ki-67 status due to the very small samples of biopsy tissue and it is difficult to reflect the overall heterogeneity of the whole tumor. Therefore, there is an urgent need for a non-invasive, convenient, and comprehensive method for preoperative prediction of the level of Ki-67 expression.

Magnetic resonance imaging (MRI) allows better depiction of tumor due to the high soft tissue resolution and thus has been widely used for preoperative evaluation of the tumors in practice. Radiomics is an emerging method for medical image analysis, which can extract high-dimensional and quantitative features from routine radiological imaging (9). During the past few years, there have been several studies on the application of MRI based-radiomics to predict Ki-67 proliferation status in malignant tumors. For instance, Li et al. (10) and Ye et al. (11) found that the radiomics texture features based on dynamic contrast-enhanced magnetic resonance imaging (DCE-MRI) can predict the Ki-67 expression in liver cancer. Ma et al. (12) demonstrated that the quantitative radiomics features extracted from DCE-MRI are associated with Ki-67 status in breast cancer. In the field of sinonasal malignancy, so far only one study by Bi et al. (13) used radiomics analysis to predict the status of Ki-67, and they found that the constructed multiparametric MRI-based radiomics signature (RS) can effectively evaluate Ki-67 expression with AUC and accuracy of 0.852% and 86.3%, respectively. However, in all the studies mentioned above, radiomics analysis was undertaken based on the conventional handcrafted (HC) features.

More recently, with the increasingly popular use of computer-aided detection systems and artificial intelligence technology in oncologic imaging, deep learning (DL) has been preliminarily applied for image pattern recognition. It can provide more abundant texture and biological information of lesions. Actually, training a DL model commonly requires immense amounts of labeled data before the predictive value in clinical practice can be reached. However, on the one hand, the low incidence rate of sinonasal malignancy makes it difficult to enroll a large number of patients for DL analysis, and on the other hand, labeling the big data is a laborious and time-wasting work. To overcome these limitations, deep transfer learning (DTL) has been applied in the clinical trial. By pretraining a model to explore the critical features, pretrained learning is then applied in DTL to a related image task; subsequently, processes of fine-tuning can adjust the network for the new feature detection task (14, 15). With regard to the use of MRI-based DTL as an optional way to predict Ki-67 expression in malignancy, to date, there has been only one study reported by Liu et al. (16). In their study on 328 breast cancers, DTL-based radiomics models were established for preoperative prediction of Ki-67 status using multiparametric MRI and yielded better predictive efficacy (AUC = 0.875 in the validation dataset).

Since in previous studies radiomics analysis were always separately conducted using either HC features or DTL features, the advantages and limitations of the two types of image features have not been well investigated. In the current study, based on different machine learning (ML) models, we made an attempt to construct and validate a model that integrates the HC features and DTL features obtained from multiparametric MRI to estimate the Ki-67 status in SNSCC.

## 2 Patients and materials

### 2.1 Patients

**Figure 1:**
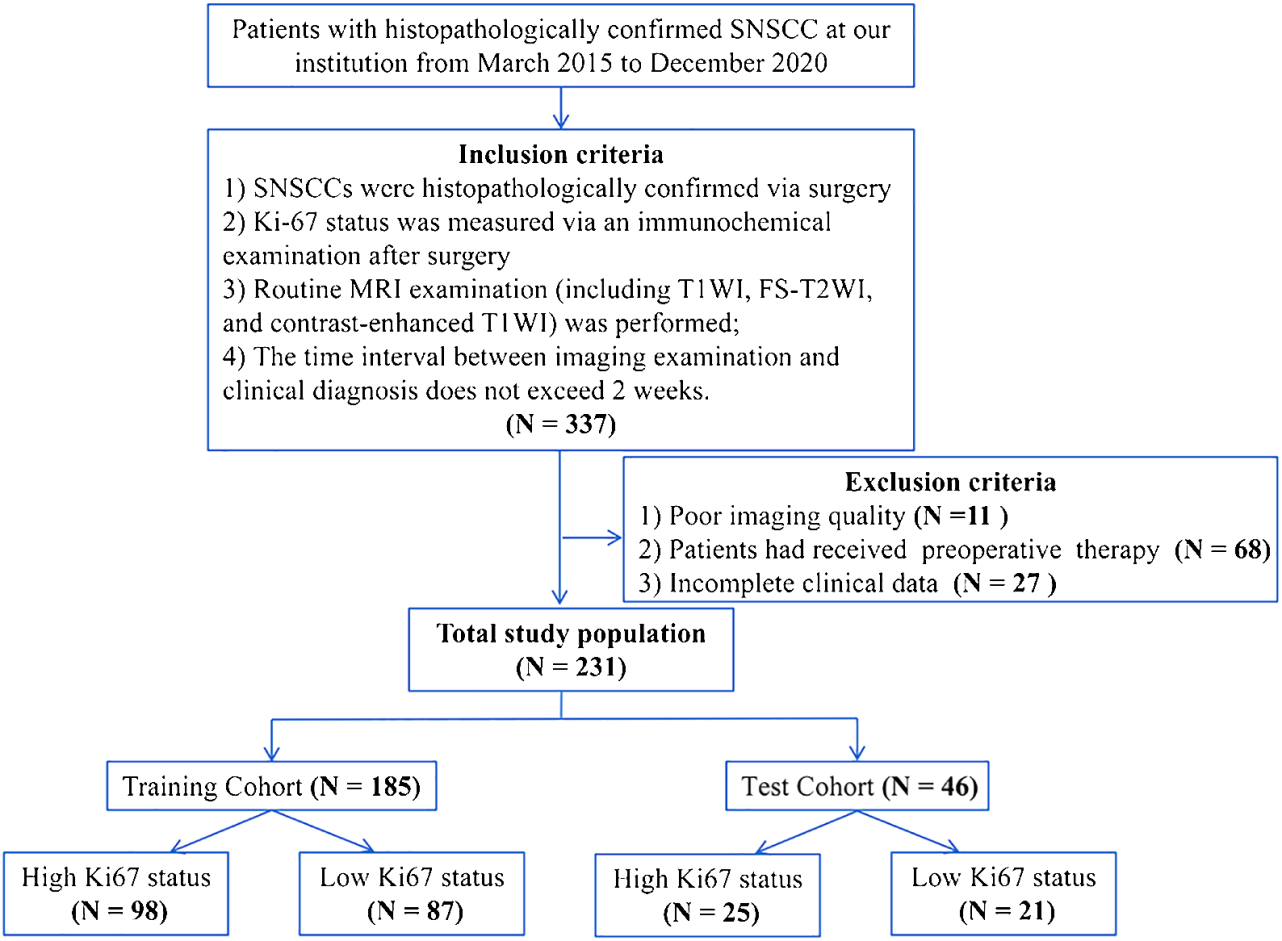
The inclusion and exclusion criteria of the patients.

This study was approved by the Institutional Review Board of our hospital. In this retrospective study, we reviewed 337 cases of histopathologically confirmed SNSCC at our institution from March 2015 to December 2020. The inclusion and exclusion criteria of the patients are shown in **Figure 1**. Finally, a total of 231 patients (169 men, 62 women, mean age 57.49 ± 14.23 years old) were included. The patients were randomly divided into training and testing cohorts at a ratio of 8:2. Clinical indexes including gender, age, epistaxis, and clinical stage were collected.

### 2.2 Ki-67 index measurement

The Ki-67 status was determined by immunohistochemical from postoperative mass excision material samples. A Ki-67 protein antibody was used to perform the immunohistochemical staining. The Ki-67 index was scored according to the proportion of Ki-67-positive cells. The mean value of the Ki-67 index was 53.29% ± 22.04% (range: 5%–90%; median: 55%) in our study. The cutoff value of 50% was used for determining the high Ki-67 index (Ki 67 ≥50%) and low Ki-67 index (Ki-67<50%).

### 2.3 MRI image acquisition

All patients underwent MR examination using a 3T scanner (Magnetom Verio or Prisma; Siemens Healthcare, Erlangen, Germany) with a 12-channel head and neck coil. Axial fat-saturated T2-weighted imaging (FS-T2WI) was firstly acquired. Then, diffusion-weighted imaging (DWI) was performed using a high-resolution DWI system (b values = 0, 1,000 s/mm^2^). The apparent diffusion coefficient (ADC) map was derived from DWI. After the intravenous administration of gadolinium-diethylenetriamine pentaacetic acid (Magnevist, Bayer Schering, Berlin, Germany), axial fat-saturated contrast-enhanced (CE) T1WI scans were obtained. The detailed parameters are shown in **Table 1**.

**Table 1:** Parameters of the enrolled MR sequences.

	T2WI	DWI	CE-T1WI
Sequence	Turbo spin-echo	Readout-segmented echo-planar imaging, two-dimensional navigator-based reacquisition	Turbo spin-echo
Repetition time (ms)	4,190 (scanner 1)	3,700 (scanner 1)	4.7 (scanner 1)
Echo time (ms)	81 (scanner 1)	66 (scanner 1)	1.8 (scanner 1)
Thickness (mm)	4	4	3
Field of view (mm^2^)	230 × 230	230 × 230	230 × 230

### 2.4 MRI characteristics

As on T1-weighted imaging (T1WI), the border of the tumor was ill-defined and the signal features are non-specific, in the current study, we did not analyze the image findings on T1WI. The MRI characteristics on FS-T2WI, ADC, and CE images were reviewed independently by two radiologists [readers 1 and 2, with 5 and 8 years of work experience, respectively] on a Siemens Syngo workstation. The disagreement was resolved through further discussion with a third radiologist [reader 3, with 20 years of experience] to reach a consensus. The characteristics include (a) maximum tumor diameter (>5 cm or<5 cm), (b) margin (well-defined or ill-defined), (c) laterality (unilateral or bilateral), (d) cysts/necrosis areas within tumor (yes or no), (e) enhancement degree [moderate (enhancement approaching that of the adjacent muscle) or apparent (enhancement approaching that of the adjacent vessels)], (f) bone destruction (yes or no), (g) enlarged (short diameter >1.0 cm)/necrotic lymph node (yes or no), and (h) ADC value. When measuring the ADC value, a small circular ROI was placed on the darkest area of the lesion on the ADC map avoiding cystoid variations, hemorrhage, and necrosis areas. For each case, three ROIs were placed and the lowest ADC was retained. The size of the each ROI was 0.5 cm^2^–1 cm^2^.

### 2.5 Histopathological-clinical-image model

To assess the histopathological grade, clinical data, and MRI features, we used chi-square test to compare categorical variables, Fisher’s exact test for groups with small sample sizes, and independent samples t-test for normally distributed continuous variables. To choose the independent predictors of high Ki-67 status, univariate logistic regression (LR) analysis was used to analyze the histopathological, clinical, and MRI image features with p< 0.100, and then multivariate LR analysis (backward stepwise: Wald) was used to select the statistically significant predictors by analyzing features with p< 0.05. Finally, the factors with p< 0.05 were considered as the independent predictors and enrolled into the histopathological-clinical-image model.

### 2.6 Tumor segmentation and radiomic data preprocessing

**Figure 2:**
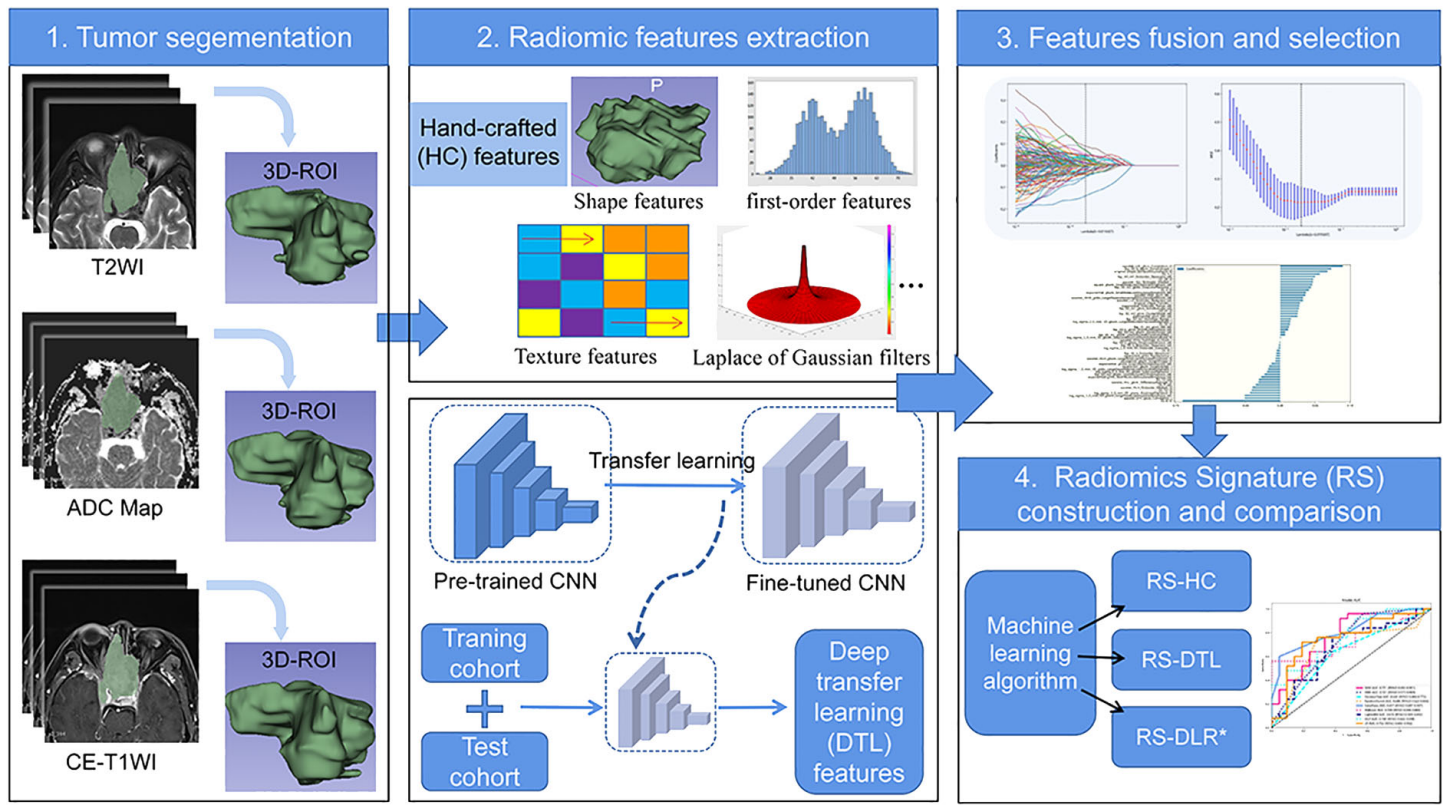
Flowchart of radiomics for predicting Ki-67 status in patients with SNSCC.

The radiomic workflow is displayed in **Figure 2**.

Tumor segmentation was conducted by two radiologists with 8 years of experience and 20 years of experience in head and neck radiology independently using the ITK-SNAP software (www.itksnap.org). The radiologists were blinded to the patient’s histopathological findings before analyzing the images. The volumes of interests (VOIs) were outlined slice by slice to cover the whole tumor avoiding obvious necrotic and cystic areas on three sequences (FS-T2WI, ADC, and CE-T1WI), respectively.

Because the range of pixel values of medical images varies under different MRI scanners, we sorted all the pixel values in each image and truncated the intensities to the range of 0.5 to 99.5 percentiles to reduce the side effect of pixel value outliers. VOIs are common with heterogeneous voxel spacing because of different acquisition protocols. The fixed resolution resampling method was applied to reduce the effect of voxel spacing variation.

### 2.7 HC radiomic feature extraction

The HC radiomic features were extracted from the image set using PyRadiomics (www.radiomics.io/pyradiomics.html), including shape features, first-order features, and textural features. Texture features included the gray-level co-occurrence matrix (GLCM), gray-level run length matrix (GLRLM), gray-level size zone matrix (GLSZM), and neighborhood gray-tone difference matrix (NGTDM) methods. Eight wavelet transformations algorithms (LLL, LLH, LHL, LHH, HLL, HLH, HHL, and HHH) and Laplace of Gaussian (LoG) filters were conducted for first-order and textural features. A total of 3,495 HC radiomics features were extracted from three MR sequences. The interoperator variability of the features was evaluated by the intraclass correlation coefficient (ICC). Features with intra-ICCs >0.75 were retained for subsequent analysis.

### 2.8 DTL feature extraction and compression

DTL features were extracted from pretrained CNN via transfer learning. In this study, ResNet50 was chosen as the pretrained CNN model. The Resnet50 model was trained on the 2012 ImageNet Large Scale Visual Recognition Challenge (ILSVRC-2012) dataset (17). The slice which had the largest tumor area was picked out to represent each patient. Then, the gray values were normalized to range [-1, 1] using min–max transformation. Next, each cropped subregion image was resized to 224 × 224 with nearest interpolation. The obtained images can be used as the model input.

Because of leak of image data, in order to better carry out the generalization, we carefully set the learning rate. We adapted cosine decay learning rate algorithm in this study. Our learning rate was presented as follows:


$$\eta_{t}^{task-spec}=\eta_{min}^{i}+\frac{1}{2}\left(\eta_{max}^{i}-\eta_{min}^{i}\right)\left(1+cos\left(\frac{T_{cur}}{T_{i}}\pi \right)\right)$$

$\eta_{min}^{i}=0$ , $\eta_{max}^{i}$ = 0.01, and $T_{i}=30$ represent the minimum learning rate, the maximum learning rate, and the number of iteration epochs, respectively. Because the backbone part adopted pretraining parameters, in order to ensure the migration effect, $T_{cur}=\frac{1}{2}T_{i}$ fine-tunes the parameters of the backbone part. Therefore, the learning rate of backbone part was as follows:


$$\eta_{t}^{backbone}=\{\begin{array}{ll} 0 & \text{if}T_{cur}\leq \frac{1}{2}T_{i}\\ \eta_{min}^{i}+\frac{1}{2}\left(\eta_{max}^{i}-\eta_{min}^{i}\right)\left(1+cos\left(\frac{T_{cur}}{T_{i}}\pi \right)\right) & \text{if}T_{cur}>\frac{1}{2}T_{i} \end{array}$$

(Hyperparameters: cross entropy was used as loss function, SGD preformed as optimizer, learning rate was initialized from 0.01, batch size was 32, training max epoch was set to 30, with early stop at 5).

In order to ensure the balance between features, we subsequently used principal component analysis (PCA) to reduce the dimension of DTL features from 2,048 to 96 to improve the generalization ability of the model and reduce the risk of over fitting.

### 2.9 Feature fusion and selection

The HC radiomics features group and compressed DTL features group were fused together to formulate the deep learning-based radiomics (DLR) features group for subsequent analysis process. All the DLR features were normalized (Z-score transformation). Then, based on the training cohort, a least absolute shrinkage and selection operator (LASSO) model with fivefold cross-validation was applied to select the most meaningful features.

### 2.10 Radiomics signature

We put each feature group into different ML algorithms to construct three RSs (RS-DLR, RS-DTL, and RS-HC). Here, we adopt nine ML algorithms including support vector machine (SVM), k-nearest neighbor (KNN), decision tree (DT), random forest (RF), extra trees (ET), XGBoost, LightGBM, multilayer perception (MLP), and LR for RS-DTL and RS-DLR, and 8ML algorithms for RS-HC. To evaluate the performance of three RSs, we compared the following indicators of RSs in the training and testing sets: the area under the receiver operating characteristic curve (AUC), sensitivity, specificity, accuracy, positive predictive value (PPV), and negative predictive value (NPV). Then, RS with the highest predictive performance was chosen as the best RS.

### 2.11 Statistical analysis

The statistical analysis were performed on SPSS (v.24.0), MedCalc (version v.19.0.4), R software (v.4.1.0), and Python (v3.7.6). p< 0.050 was regarded as statistical significance.

## 3 Results

### 3.1 Clinical and MRI characteristics

High Ki-67 expression was present in 53.0% of the training cohort and 54.3% of the testing cohort. The clinical and MRI manifestations of SNSCC in two cohorts are shown in **Table 1**. There were no significant differences in patients’ clinical, histopathological, and MRI characteristics between high- and low-Ki-67 groups in both training and testing cohorts (p > 0.050) (**Table 2**).

**Table 2:** Clinical data and conventional MRI characteristics of the patients.

Characteristic		Training cohort		*P* value	Testing cohort		*P* value
		High ki-67	Low ki-67		High ki-67	Low ki-67	
		(n= 98)	(n= 87)		(n=25)	(n=21)	
Clinical characteristics	**Gender**			0.515			0.489
	Male	74	62		19	14	
	Female	24	25		6	7	
	**Age**			0.527			0.071
	Mean ± SD (years old)	56.9 ± 14.77	57.7 ± 14.75		60.0 ± 9.96	56.4 ± 14.36	
	**Epistaxis**			0.216			0.939
	Yes	39	27		11	9	
	No	59	60		14	12	
	**Clinical stage**			0.142			0.194
	I~II	24	30		10	14	
	III	48	43		8	4	
	IV	26	14		7	3	
Histopathological characteristics	**Grade**			0.100			0.566
	I~II	55	60		16	16	
	III	43	27		9	5	
Conventional MRI characteristics	**Maximum diameter**			0.064			0.054
	>5 cm	47	30		13	5	
	<5 cm	51	57		12	16	
	**ADC value**			0.323			0.123
	Mean ± SD (×10^−^ **3 mm2/s)**	0.769 ± 0.126	0.831 ± 0.130		0.752 ± 0.108	0.851 ± 0.149	
	**Margin**			0.084			0.179
	Well-defined	20	24		6	9	
	Ill-defined	78	63		19	12	
	**Laterality**			0.550			0.988
	Unilateral	14	16		19	16	
	Bilateral	84	71		6	5	
	**Cysts/necrosis**			0.341			0.208
	yes	83	69		13	7	
	no	15	18		12	14	
	**Enhancement degree**			0.992			0.669
	Moderate	54	48		17	13	
	Apparent	44	39		8	8	
	**Bone destruction**			0.997			0.750
	Yes	80	71		18	16	
	No	18	16		7	5	
	**Enlarged/necrotic lymph node**			0.114			0.224
	Yes	27	15		7	2	
	No	71	72		18	19	

Then, we enrolled the factors with p< 0.100 into univariate LR analysis; however, the results revealed the absence of statistically significant clinical, histopathological, and MRI independent predictors for Ki-67 status. This means the histopathological-clinical-image model failed to be built.

### 3.2 Radiomics feature extraction, fusion, and selection

**Figure 3:**
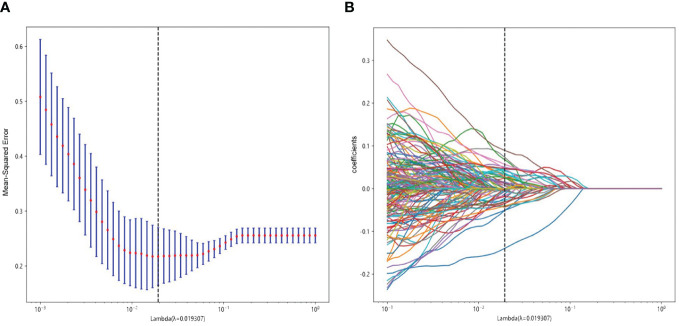
Radiomics feature selection using LASSO in the training cohort. **(A)** Radiomics feature selection. **(B)** The non-zero coefficients have been plotted.

**Figure 4:**
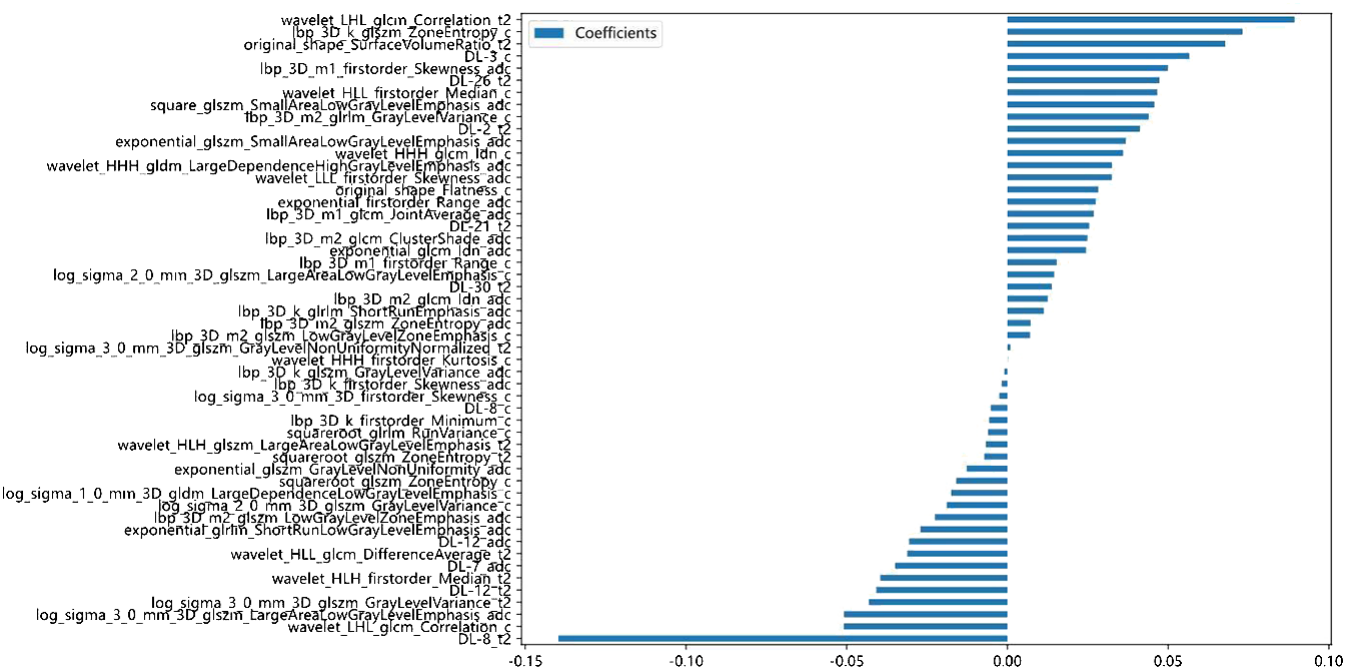
Distributions of 52 optimal features based on LASSO regression in the training cohort.

A total of 3,071 HC features with ICC >0.75 were retained for analysis. The 3,071 HC and 96 DTL features were fused together to formulate the DLR features group. Based on LASSO regression (as shown in **Figure 3**), the DLR features were reduced to 52 optimal features, which included 42 HC radiomics features (8 T2WI-based features, 16 CE-based features, and 18 ADC-based features) and 10 DTL features (6 T2WI-based features, 2 CE-based features, and 2 ADC-based features). **Figure 4** shows the distributions of 52 optimal features in the training cohort.

### 3.3 Performance of RS-DLR vs. RS-DTL vs. RS-HC

For RS-HC, the optimal classifier was the SVM algorithm, with an AUC of 0.803 (95% CI: 0.679–0.927) and accuracy, sensitivity, specificity, PPV, and NPV of 0.728, 0.880, 0.762, 0.759, and 0.824 in the test cohort, respectively.

For RS-DTL, the optimal classifier was LightGBM algorithm, with a high AUC of 0.987 (95% CI: 0.976–0.999) in the training cohort, whereas in the test cohort, the predictive ability was not as high as that in the training cohort; the AUC value was only 0.650 (95% CI: 0.487–0.812) and the values of accuracy, specificity, PPV, and NPV were all relatively low (<0.800).

For RS-DLR, the optimal classifier was the ET algorithm, with an extremely high AUC of 1 in the training cohort. In the test cohort, the predictive ability was also excellent, the AUC value was 0.817 (95% CI: 0.697–0.937), and specificity was 0.952, which were superior to those of RS-HC and RS-DTL; thus, the RS-DLR was chosen as the best RS in our study.

**Figure 5:**
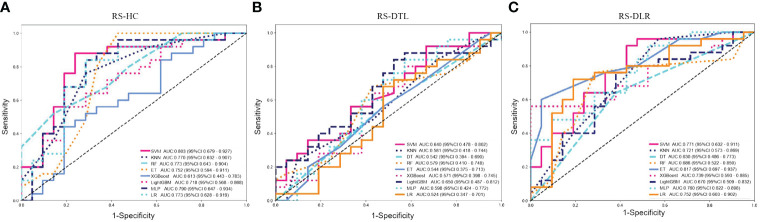
Predictive performances of **(A)** RS-HC, **(B)** RS-DTL, and **(C)** RS-DLR based on different ML classifiers in the test cohort (SVM, support vector machine; KNN, k-nearest neighbor; DT, decision tree; RF, random forest; ET, extra trees; MLP, multilayer perception; LR, logistic regression).

However, we also observed that the AUC of RS-DLR in the test cohort failed to show statistically significant difference from the that of RS-HC (p > 0.050) (**Table 3**), which means that both the RS-HC and RS-DLR can achieve excellent predictive ability. **Figure 5** shows the prediction performances of RS-HC, RS-DTL, and RS-DLR based on different ML classifiers, respectively.

**Table 3:** The predictive performances of three RSs in the training and test cohorts.

RS type	Cohort	Optimal classifier	AUC	95% CI	Accuracy	Sensitivity	Specificity	PPV	NPV
RS-HC	Training	SVM	0.882	0.828–0.936	0.832	0.847	0.851	0.807	0.868
	Test	SVM	0.803	0.679–0.927	0.728	0.880	0.762	0.759	0.824
RS-DTL	Training	LightGBM	0.987	0.976–0.999	0.956	0.990	0.920	0.933	0.988
	Test	LightGBM	0.650	0.487–0.812	0.630	0.840	0.476	0.643	0.611
RS-DLR	Training	ExtraTrees	1.000	/	1.000	1.000	1.000	1.000	1.000
	Test	ExtraTrees	0.817	0.697–0.937	0.673	0.600	0.952	0.667	0.688

RS, radiomics signature; AUC, area under curve; PPV, positive predictive value; NPV, negative predictive value.

## 4 Discussion

Ki-67 is an important indicator related to tumor heterogeneity and cell proliferation. Extensive literatures (5, 18) on cell cycle analysis showed that Ki-67 was helpful in predicting the tumor prognosis and thus has been applied widely in clinical decision-making for tumor treatment.

In the current study, an investigation of the proliferation of Ki-67 in SNSCC, which combine the HC and DTL features from multiple MR sequences, revealed the ET and ResNet50 algorithm-based DLR model to be required for the highest AUC result, while the pathological grade, clinical, and conventional MRI characteristics did not show predictive value for Ki-67 status in SNSCC.

MRI has been widely used for diagnosing the sinonasal tumors. However, due to the lack of specific image features, it is difficult to predict the expression of Ki-67 status on conventional MRI. A research by Xiao et al. (8) has proposed that the combined use of quantitative dynamic contrast-enhanced MRI and intravoxel incoherent motion model of DWI was helpful for predicting Ki-67 status of sinonasal cancer. However, in view of the significantly prolonged image acquisition time and quite complex modeling process, this image approach was not easy to widely popularize.

Radiomics, which is an evolving field for assessment of disease, can extract high-throughput features from medical images and assess the tumor biology. The use of radiomics in predicting the Ki-67 status has been reported by many researchers (10–13). In head and neck squamous cell carcinoma, Zheng et al. constructed and validated the computed tomography (CT)-based radiomics nomogram model to predict the Ki-67 expression level (19). However, given the high degree of heterogeneity of the malignant tumor, more and more controversies remain as to whether the traditional HC features are comprehensive and precise enough for evaluating the biological characteristics of whole tumor.

In recent years, developments in the field of computer-aided signal processing and the expansion of computing power with the latest high-speed graphics processors make DL-based radiomic analysis primarily used for tumor recognition and diagnosis. In order to better train or finetune the DL model, many modified or advanced DL models have been developed, including visual geometry group (VGG)16, VGG19, and ResNet50. Among them, the ResNet network, which is modified from the VGG19 network and constructed by adding residual blocks through the short-circuit mechanism, can not only save the operational time but also reduce the learning difficulty of the network. Previous studies (20, 21) have found that the ResNet50 model was the best architecture framework with the highest accuracy and efficiency for the image classification task. Danala et al. (14) proposed that the pretrained ResNet50 model-based DTL feature can yield significantly higher AUC than that of the traditional HC feature for characterizing the malignancies (p< 0.01).

In our study, we also trained the DL method on the ResNet50 for DTL features extracting and applied to fine-tune on ResNet50 model for prediction task. After comparison of the 9 ML algorithm, LightGBM was the best classifier for DTL-RS. For HC-RS, the ML algorithm of SVM was the best classifier with the highest AUCs in the training and test sets. After integrating HC with DTL features, the classifier of ET owned the best predictive ability than other algorithms for DLR-RS. Actually, ET was initially derived from the traditional algorithm of DT in 2005 by adding some innovative algorithm steps and improvement in DT. On the one hand, it increases the randomness of the DT algorithm, on the other hand, it improves the accuracy of the suboptimal solution and calculation flexibility. Maier et al. (22) found that ET owned better performance than SVM for voxel-wise classification because it turned out to be easy to tune and not sensitive to the selection of the training data. In the current study, the ET algorithm-based DLR-RS performed significantly better than LightGBM algorithm-based DTL-RS and SVM algorithm-based HC-RS in the training cohort. In the test set, the AUC of DLR-RS was also superior to HC-RS and DTL-RS. A previous work by Bo et al. (23) used multiparametric MRI to distinguish brain abscess from cystic glioma and showed that DTL features combined with HC features could contribute to a significantly higher accuracy than HC and DTL features alone. Another study by Hu et al. (24) using DWI to diagnose breast cancer demonstrated that the diagnostic efficiency of the HC-DL-fusion classifier was significantly higher than the HC-based classifier and slightly higher than the DL-based classifier. These outcomes, in general, indicated that the fusion model yields more biological information about tumor than a single type of radiomic features. However, in our study, we observed that in the test dataset, there was no significant difference of the performance between DLR-RS and HC-RS (both AUCs > 0.8), which means both the HC and DLR model showed favorable predictive efficacy in patients with SNSCC.

In the current study, we did not establish the histopathological-clinical-image model because all the histopathology, clinical, and MRI characteristics lack statistical significance for predicting Ki-67 status. Even though the SNSCC tumors of high Ki-67 proliferation status tended to show higher histopathological grade, larger maximum diameter, and ill-defined margin, univariate and multivariate LR analyses showed no independent predictor was observed. A similar result was also detected by Bi et al. (13); in their study on 128 patients with different pathological types of sinonasal malignancies, no independent predictor of high Ki-67 status was found based on age, gender, signal feature, tumor margin, size, level of enhancement, etc.

Our study has certain limitations. Firstly, the performance of DTL-RS in the test cohort was not high and we have noticed that in some studies (22, 23) the CNN model of VGG-19 was chosen for DTL feature extraction instead of Resnet50, as they believed that VGG-19 can better focus on the details of the tumor region; thus, further effort on VGG-19 is needed to enhance the performance of the DTL classifier and integrated RS-DLR. Secondly, because of the rarity of SNSCC, we used a relatively small sample from one institution; the multicenter, large-sample experiments will be beneficial in order to validate the applicability of the model. Thirdly, in general, the predictive capacity of RS-DLR still remains poorly understood; the advanced feature fusion method also needs to be studied to improve the model accuracy in the future.

To our knowledge, this is the first report to focus on the associations of multiparametric MRI-based integrated DLR-RS, clinical risk factors, and MRI manifestations, with Ki-67 expression in patients with SNSCC. Our results demonstrated that based on the ET classifier, the integrated RS-DLR could represent an opportunity to advance precise prediction for Ki-67 status and provide a reference for individualized treatment plans in SNSCC. Further predictive value remains to be promoted by subsequent studies.

## Data availability statement

Requests to access the datasets should be directed to linnaierbaby@qq.com.

## Ethics statement

The studies involving humans were approved by the ethics committee of Eye and ENT Hospital of Fudan University. The studies were conducted in accordance with the local legislation and institutional requirements. Written informed consent for participation was not required from the participants or the participants’ legal guardians/next of kin in accordance with the national legislation and institutional requirements.

## Author contributions

NL: Funding acquisition, Investigation, Writing – original draft, Writing – review & editing. YQS: Data curation, Formal analysis, Investigation, Validation, Writing – review & editing. MY: Conceptualization, Data curation, Formal analysis, Investigation, Resources, Validation, Writing – review & editing. LW: Investigation, Writing – review & editing. YS: Supervision, Writing – review & editing.
